# Meningitic *Escherichia coli* Induction of ANGPTL4 in Brain Microvascular Endothelial Cells Contributes to Blood–Brain Barrier Disruption via ARHGAP5/RhoA/MYL5 Signaling Cascade

**DOI:** 10.3390/pathogens8040254

**Published:** 2019-11-22

**Authors:** Lu Liu, Jixuan Li, Dong Huo, Zhong Peng, Ruicheng Yang, Jiyang Fu, Bojie Xu, Bo Yang, Huanchun Chen, Xiangru Wang

**Affiliations:** 1 State Key Laboratory of Agricultural Microbiology, College of Veterinary Medicine, Huazhong Agricultural University, Wuhan 430007, China; cofeandsalt@163.com (L.L.); lijixuan313@163.com (J.L.); hdhznydx@163.com (D.H.); pengzhong@mail.hzau.edu.cn (Z.P.); yangrc320@aliyun.com (R.Y.); fujiyang921021@hotmail.com (J.F.); wulixubojie@hotmail.com (B.X.); 13100637110@163.com (B.Y.); 2 Key Laboratory of Preventive Veterinary Medicine in Hubei Province, The Cooperative Innovation Center for Sustainable Pig Production, Wuhan 430007, China; 3 Key Laboratory of Development of Veterinary Diagnostic Products, Ministry of Agriculture of the People’s Republic of China, Wuhan 430007, China; 4 International Research Center for Animal Disease, Ministry of Science and Technology of the People’s Republic of China, Wuhan 430007, China

**Keywords:** bacterial meningitis, BBB disruption, ANGPTL4, ARHGAP5, RhoA, MYL5

## Abstract

Bacterial meningitis is currently recognized as one of the most important life-threatening infections of the central nervous system (CNS) with high morbidity and mortality, despite the advancements in antimicrobial treatment. The disruption of blood–brain barrier (BBB) induced by meningitis bacteria is crucial for the development of bacterial meningitis. However, the complete mechanisms involving in the BBB disruption remain to be elucidated. Here, we found meningitic *Escherichia coli* induction of angiopoietin-like 4 (ANGPTL4) in brain microvascular endothelial cells (BMECs) contributes to BBB disruption via ARHGAP5/RhoA/MYL5 signaling cascade, by the demonstration that ANGPTL4 was significantly upregulated in meningitis *E. coli* infection of BMECs as well as mice, and treatment of the recombinant ANGPTL4 protein led to an increased permeability of the BBB in vitro and in vivo. Moreover, we found that ANGPTL4 did not affect the expression of tight junction proteins involved in BBB disruption, but it increased the expression of MYL5, which was found to have a negative role on the regulation of barrier function during meningitic *E. coli* infection, through the activation of RhoA signaling pathway. To our knowledge, this is the first report demonstrating the disruption of BBB induced by ANGPTL4 through the ARHGAP5/RhoA/MYL5 pathway, which largely supports the involvement of ANGPTL4 during meningitic *E. coli* invasion and further expands the theoretical basis for the mechanism of bacterial meningitis.

## 1. Introduction

Bacterial meningitis is recognized as one of the most important life-threatening infections of the central nervous system (CNS) with high morbidity and mortality, despite the advancements in antimicrobial treatment [1]. Even in developed areas of the world with good medical treatment, the mortality is still up to 30%. The incidence of bacterial meningitis is about five cases per 100,000 adults per year in developed countries and may be 10 times higher in less developed countries [2]. It estimates that the case fatality rates of the disease range from 5%–25%, and approximately 25%–50% of survivors sustain neurologic sequelae [3]. The blood–brain barrier (BBB) is composed of highly specialized brain microvascular endothelial cells (BMECs), pericytes, and astrocyte endfeet, separates brain tissue from the circulating blood, and maintains homeostasis of the neuronal environment [4,5,6]. The BMECs are interconnected by tight junctions which consist of cytoplasmic zonula occludens (ZO) proteins and various transmembrane proteins such as Occludin and Claudins [7,8]. Despite its highly restrictive nature, certain bacterial pathogens, such as *Streptococcus pneumoniae*, *Neisseria meningitidis*, *Haemophilus influenzae*, and *Escherichia coli*, are able to gain entry into the CNS resulting in serious disease [9,10,11,12,13,14]. Scientifically, the commencement of bacterial meningitis initiates from blood-borne bacteria infiltration into the BBB, then getting entrance into the CNS. The hallmark events within the pathophysiology of bacterial meningitis include the increased cytokines/chemokine levels, deteriorating endothelium barrier integrity through adherens junctions and tight junctions deformation, and BBB dysfunction [15]. Particularly, disruption of BBB tight junctions has been well documented in CNS infections, which is considered to be a pathological condition in the development of the diseases [16,17]. However, the complete mechanism of BBB disruption induced by meningitis-causing bacteria during infection still remains to be elucidated.

Angiopoietin-like 4 (ANGPTL4) is a fasting-induced inhibitor of lipoprotein lipase (LPL) and a regulator of plasma triglyceride metabolism [18]. Known as a downstream target of the ligand-activated transcriptional factors peroxisome proliferator-activated receptor γ (PPARγ) and PPARα, ANGPTL4 plays important roles in lipid and glucose metabolism [19]. ANGPTL4 can be cleaved on secretion, and its N-terminal and C-terminal fragments play discrete biological functions [19]. A study showed that nANGPTL4 is an orphan ligand that mainly controls lipid metabolism and cANGPTL4 binds—e.g., β-integrins, VE-cadherin, and claudin-5—to induce vascular leakage and tumor progression [19]. An increasing number of studies have indicated the possible involvement of ANGPTL4 in the development of cardiovascular diseases [20,21,22], inflammatory diseases [23,24,25,26], cancers [27,28,29], etc. However, there are still no reports on the role of ANGPTL4 in bacterial meningitis.

Our previous RNA-sequencing work have found that the ANGPTL4 gene was significantly upregulated (*p* ≤ 0.05) in human BMECs (hBMECs) in response to meningitic *E. coli* infection [30], speculating a potential role of this gene during meningitic *E. coli* invading the BBB. In the current work, we investigated the generation as well as biological role of ANGPTL4 in meningitic *E. coli*-induced disruption of the BBB. We demonstrated preliminarily that meningitic *E. coli* induced the expression of ANGPTL4 through the activation of PPARβ/δ signaling pathway, and ANGPTL4 contributed to the infection-mediated BBB disruption via the ARHGAP5/RhoA/MYL5 signaling cascade, affecting the actin cytoskeleton. Characterizing the biological roles of these meningitic *E. coli*-responsive host genes and signaling in BMECs shall further expend the current knowledge on meningitis-causing *E. coli* invasion of the BBB.

## 2. Results

### 2.1. Meningitic E. coli Induced the Expression of ANGPTL4 through the Activation of PPAR Signaling

**Figure 1:**
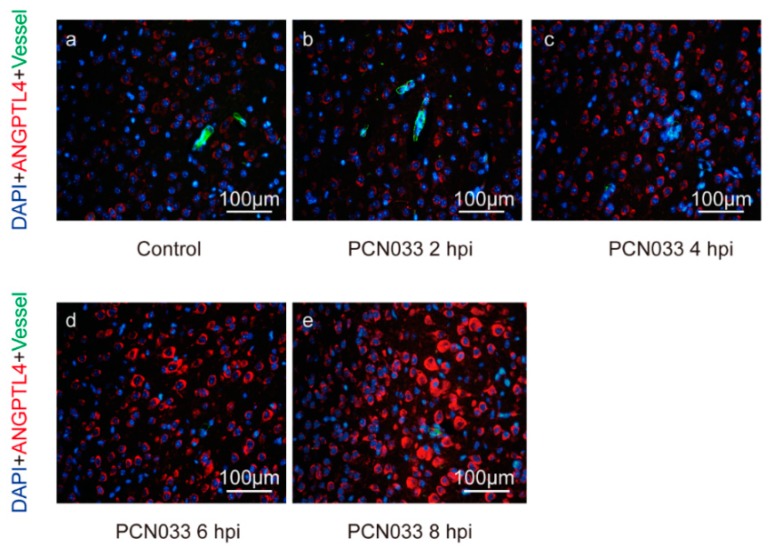
Indirect immunofluorescence of ANGPTL4 in infected mouse brains. The images show the expression alteration of ANGPTL4 in mouse brains at different time points after a challenge of meningitic *E. coli* PCN033. hpi—hours post infection. ANGPTL4 is shown in red, the blood vessels are shown in green, and DAPI (4′,6-diamidino-2-phenylindole) indicates the cell nucleus. The scale bar indicates 100 μm.

**Figure 2:**
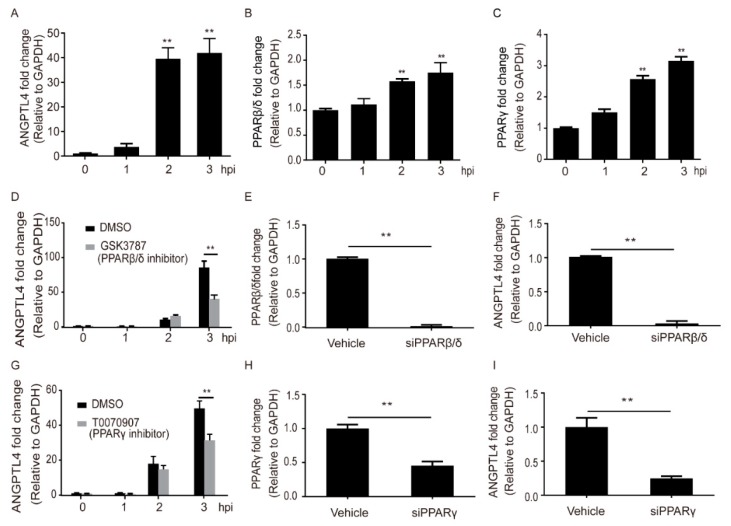
Meningitic *E. coli*-induced upregulation of ANGPTL4 in human brain microvascular endothelial cells (hBMECs) dependent on the activation of PPAR signaling. The panels (**A**), (**B**), and (**C**) indicate the expression of ANGPTL4, PPARβ/δ, and PPARγ in hBMECs after *E. coli* infection by qPCR. Panels (**D**) and (**G**) show the expression of ANGPTL4 in response to the infection with/without inhibition of PPARβ/δ or PPARγ. Panels (**E**) and (**H**) show the interfere efficiency of PPARβ/δ and PPARγ via the siRNA approaches. Panels (**F**) and (**I**) show the expression of cellular ANGPTL4 after knocking-down of PPARβ/δ or PPARγ. ** indicates extremely significant (*p* < 0.01). Data are presented as mean + standard deviation (mean + SD).

Via immunofluorescence (IF) assay, we demonstrated that the expression of ANGPTL4 protein was significantly increased in mouse brains along with the infection of meningitic *E. coli* (Figure 1). In vitro, after the challenge of meningitic *E. coli* strain, we observed a significant and time-dependent increase of ANGPTL4 in hBMECs, with a sharp increase emerging at 2 hours post infection (hpi) (Figure 2A). ANGPTL4 was canonically regulated via the PPAR-associated pathways [19], and we next investigated the possible involvement of PPAR transcriptional factors in hBMECs upon the infection. As the qPCR results show in Figure 2B,C, both transcription of PPARβ/δ and PPARγ increased significantly and displayed a time-dependent manner. We further tested their contributions in the induction of ANGPTL4 by using their specific inhibitors and showed that the PPARβ/δ inhibitor GSK3787 as well as PPARγ inhibitor T0070907 could significantly attenuate the infection-induced upregulation of ANGPTL4 (Figure 2D,G). Moreover, we knocked down the expression of PPARβ/δ or PPARγ in hBMECs using Small interfering RNA (siRNA) (Figure 2E,H) and found that either the PPARβ/δ knockdown or the PPARγ knockdown significantly reduced the ANGPTL4 expression in hBMECs (Figure 2F,I), which largely supported the notion that meningitic *E. coli* infection induced the upregulation of ANGPTL4 through the PPARβ/δ- and PPARγ-mediated signaling.

### 2.2. ANGPTL4 Aggravated the Disruption of BBB without Affecting Vitality of the hBMECs

**Figure 3:**
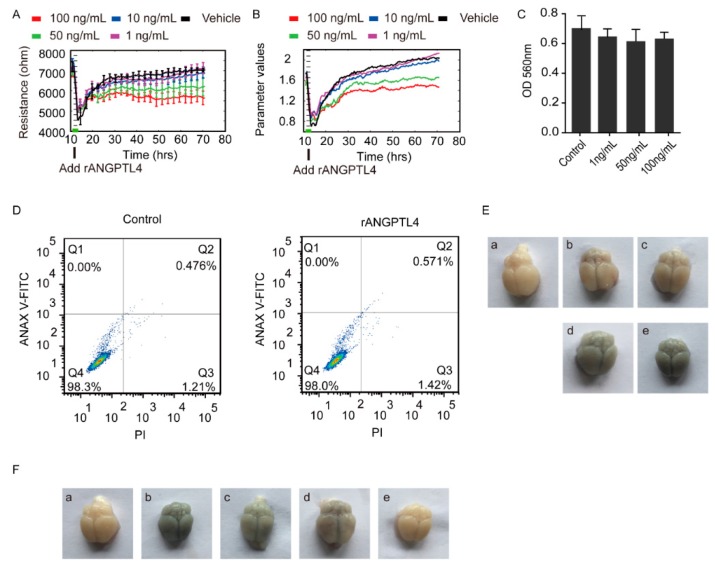
Effects of rANGPTL4 on the barrier function of hBMECs monolayer. Panels (**A**) and (**B**) indicate the effects of rANGPTL4 on the barrier function of hBMECs in vitro by the Electric Cell-Substrate Impedance Sensing (ECIS) system, and the data are presented as mean ± SD (panel A). Panel (**C**) shows the possible cytotoxicity of rANGPTL4 on hBMECs via MTT assay. Panel (**D**) reveals the possible influence of rANGPTL4 on apoptosis of hBMECs. Panel (**E**) shows the in vivo blood–brain barrier (BBB) permeability of the mice challenged with different doses of rANGPTL4 (a: PBS; b: 10 ng; c: 20 ng; d: 50 ng; e: 100 ng). Panel (**F**) exhibits the permeability of BBB in mice during *E. coli* infection with pretreatment of several PPAR pathway inhibitors (a: PBS; b: PBS+ *E. coli* challenge; c: GSK3787+ *E. coli* challenge; d: T0070907+ *E. coli* challenge; e: GSK3787+ T0070907+ *E. coli* challenge).

By using the Electric Cell-Substrate Impedance Sensing (ECIS) approach, we found the recombinant ANGPTL4 (rANGPTL4) protein exhibited an obvious barrier disruption effect on the hBMECs monolayer, by the demonstration of a dose-dependent decrease on the transendothelial electric resistance (TEER) of the hBMECs with the treatment of rANGPTL4, compared with the vehicle-treated control (Figure 3A,B). We additionally demonstrated that this disruption of the barrier function was not resulted from the destruction of cell vitality because the MTT assay showed no obvious cytotoxicity on the monolayer hBMECs in response to different concentrations (1 ng/ml, 50 ng/ml, 100 ng/ml) of rANGPTL4 (Figure 3C), and also the flow cytometry assays did not reveal the apoptosis of hBMECs in response to rANGPTL4 treatment (Figure 3D). Moreover, we tested this potential BBB disruptive role of rANGPTL4 in vivo via Evans blue infiltration assay following the tail vein injection of rANGPTL4 in mice and observed that the rANGPTL4 treatment led to an increasing infiltration of the Evans blue dye in the brains along with the increased dose of rANGPTL4 treatment, compared with the PBS-treated control (Figure 3E). This in vivo data further supports the contributive role of ANGPTL4 to the disruption of the BBB.

Since we have demonstrated meningitic *E. coli*-induction of ANGPTL4 through activating PPAR signaling, we further tested this contributive role of ANGPTL4 by blocking the PPAR signaling. As shown in Figure 3F, meningitic *E. coli* infection indeed led to an effective infiltration of the Evans blue dye into the brain (Figure 3F, Panel B vs. Panel A). However, less Evans blue dye was observed in brains of the mice receiving either GSK3787 (PPARβ/δ inhibitor) or T0070907 (PPARγ inhibitor). Particularly, there was nearly no Evans blue dye being observed in brains from the mice pretreated with both GSK3787 and T0070907 (Figure 3F, Panel C to Panel E). These data once again largely evidenced that the PPAR-signaling-determined production of ANGPTL4 exerted important roles in mediating the BBB disruption in response to meningitic *E. coli* infection. 

### 2.3. ANGPTL4 Did Not Affect Expression of the Tight Junction Proteins as Well as Cytokines Production

To explore the mechanism for ANGPTL4-mediated BBB disruption during meningitic *E. coli* infection, possible factors involved in endothelium activation were detected first through qPCR. The result revealed that the transcriptional levels of EGR-1, E-selectin, ICAM-1, and VEGFA were not significantly affected by the treatment of rANGPTL4 (Figure S1A). Since the BBB disruption would likely be developed by either the direct, tight junction breakdown or the proinflammatory factors-mediated indirect damage, we subsequently evaluated the effects of rANGPTL4 treatment on the expression of these tight junction proteins and proinflammatory cytokines. As shown, the expression of tight junction proteins including ZO-1, Occludin, and Claudin-5 were not affected by rANGPTL4 through Western blotting as well as densitometric analyses (Figure S1B,C). Likewise, the proinflammatory cytokines such as IFN-γ, IL-1β, TNF-α, IL-6, and IL-8 were not significantly induced by rANGPTL4 treatment via using electrochemiluminescence assays (Figure S1D). 

We additionally tested these observations by comparing the wildtype hBMECs and the ANGPTL4-overexpressed hBMECs. As shown, although the ANGPTL4 has been successfully overexpressed (Figure S2A), we still did not observe any expression differences of the tight junction proteins as well as the proinflammatory cytokines in the overexpressed cells, compared with the control cells (Figure S2B–D). Taken together, these findings largely supported that ANGPTL4 did not work directly to affect the tight junction proteins and the proinflammatory factors production. There are probably other novel mechanisms or pathways involving the ANGPTL4-mediated BBB disruption.

### 2.4. MYL5 and ARHGAP5 Were Differentially Expressed in Response to rANGPTL4 Treatment

**Figure 4:**
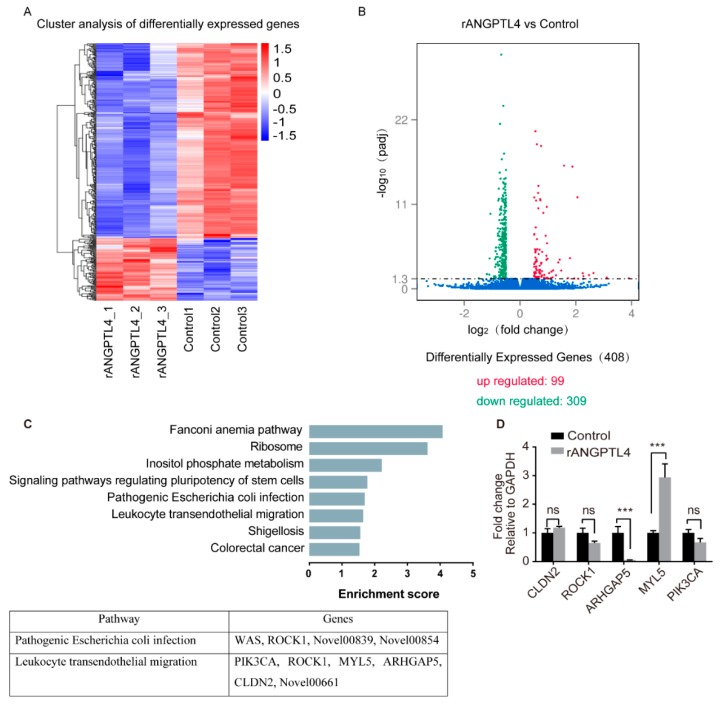
Identification of pathways targeted by ANGPTL4 using RNA-sequencing. Panel (**A**) shows the heat map of the sequencing groups. Panel (**B**) displays the volcano plots of these identified differentially expressed genes (DEGs) between rANGPTL4-treated cells and the control cells. Panel (**C**) shows the DEGs-enriched KEGG pathways as well as genes involved in two specified pathways. Panel (**D**) displays the qPCR results of the partial DEGs involved in pathogenic *E. coli* infection pathway and leukocyte transendothelial migration pathway.

To find out the effectors contributing to the ANGPTL4-induced barrier function disruption, RNA-sequencing was next performed by using the total RNAs extracted from the rANGPTL4 or PBS-treated hBMECs. Herein, we identified 99 upregulated mRNAs as well as 309 downregulated mRNAs (*p* ≤ 0.05) in ANGPTL4-treated cells, compared with the control cells (Figure 4A,B). The KEGG pathway enrichment analysis was next performed on these differentially expressed genes (DEGs), and the results revealed that DEGs identified herein were involved in several KEGG pathways, and those participating in fanconi anemia pathway, ribosome, inositol phosphate metabolism, signaling pathways regulating pluripotency of stem cells, pathogenic *E. coli* infection, leukocyte transendothelial migration, shigellosis, and colorectal cancer possessed the lowest *p*-value (Figure 4C; Table S1 in Supplementary Materials). Among these significantly enriched pathways, the pathways of “pathogenic *E. coli* infection” as well as “leukocyte transendothelial migration” were of particular concern herein.

Among these 408 DEGs identified, four genes (WAS, ROCK1, and two genes with unknown functions) and six genes (PIK3CA, ROCK1, MYL5, ARHGAP5, CLDN2, and one gene with unknown function) were enriched to the “pathogenic *E. coli* infection” and “leukocyte transendothelial migration” pathways, respectively (Figure 4C; Table S1 in Supplementary Materials). We next evaluated the expression alteration of several genes from these two pathways and observed the significant upregulation of MYL5 as well as significant downregulation of ARHGAP5 in hBMECs in response to rANGPTL4 treatment (Figure 4D), suggesting the possibility of these two genes participating in the ANGPTL4-mediated BBB disruption.

### 2.5. MYL5 Contributed to ANGPTL4-Induced Barrier Disruption of the hBMECs

**Figure 5:**
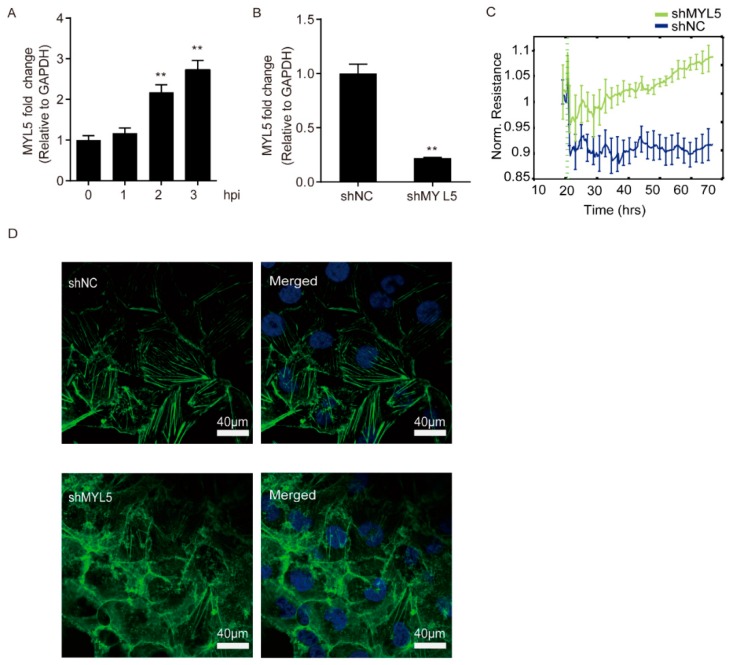
Potential effects of MYL5 on the barrier function of the hBMECs. Panel (**A**) shows the expression of MYL5 in hBMECs at different hours post infection (hpi), as detected by qPCR. Panel (**B**) shows the successful knockdown of MYL5 in hBMECs via shRNA approach. ** indicates *p* < 0.01. Panel (**C**) shows the TEER values of both MYL5-interfered cells and the control cells, as monitored by ECIS system. Data are presented as mean ± SD herein. Panel (**D**) shows the cytoskeleton alteration in hBMECs with MYL5 knocking-down by indirect immunofluorescence. The scale bar indicates 40 μm.

Based on the sequencing data, we additionally investigated the expression alteration of MYL5 in hBMECs at different time points and observed that the MYL5 was significantly increased in hBMECs along with the infection (Figure 5A). We next successfully knocked-down the expression of MYL5 in hBMECs by shRNA approach (Figure 5B) and tested its potential effects on the barrier function of the monolayer cells. As the ECIS assay shows in Figure 5C, the MYL5 knockdown monolayer cells exhibited significantly higher TEER values than that of the control cells, indicating an enhanced barrier function of the monolayer cells. We additionally observed the possible morphological change of the monolayer hBMECs after MYL5 knockdown, and found that the MYL5 knockdown cells turned to be more congested with their adjacent cells and their cytoskeleton became more closely mingled and intertwined compared with the control cells, revealing the formation of a more serried and intensive barrier of the monolayer MYL5 knocking-down cells (Figure 5D).

### 2.6. ARHGAP5/RhoA Signaling Facilitated the ANGPTL4 Regulation of MYL5 Expression in hBMECs 

**Figure 6:**
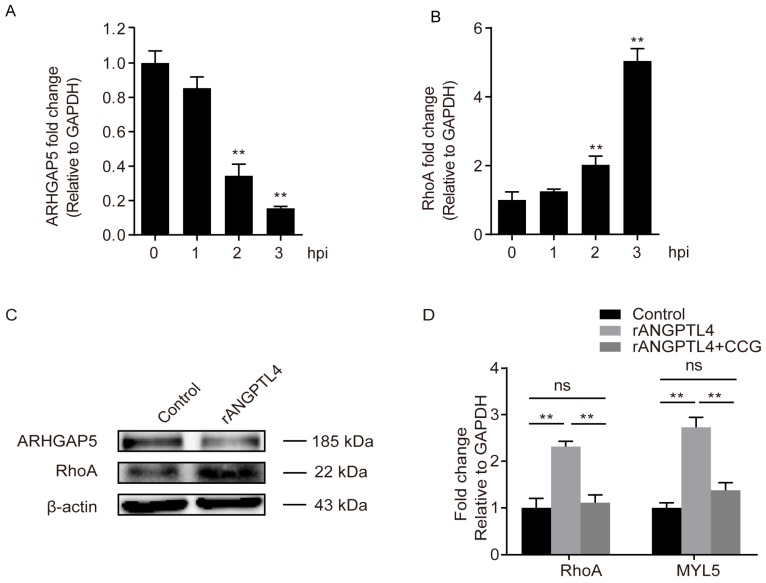
ARHGAP5/RhoA signaling mediated the ANGPTL4 regulation of MYL5 in hBMECs. Panels (**A**) and (**B**) show the time-dependent expression alteration of ARHGAP5 and RhoA in hBMECs in response to the infection, respectively, as detected by qPCR. Panel (**C**) shows the Western blotting of ARHGAP5 and RhoA expression in response to the ANGPTL4 treatment. Panel (**D**) shows the expression of RhoA as well as MYL5 in hBMECs in response to ANGPTL4, with/without pretreatment of RhoA inhibitor CCG (100 μM). ** indicates *p* < 0.01. Data are presented as mean + SD.

Another ANGPTL4-responsive DEGs, ARHGAP5, was also identified in our RNA-sequencing and qPCR results (Figure 4D). As shown, the ARHGAP5 exhibited a significantly decreased expression in hBMECs along with the infection (Figure 6A), while in contrast, the RhoA exhibited an increasing expression in response to the infection (Figure 6B), which is exactly consistent with the concept that ARHGAP5 could negatively regulate RhoA [31]. Moreover, we demonstrated by Western blotting that the treatment of ANGPTL4 led to a significant reduction of ARHGAP5 expression, while it significantly increased the expression level of RhoA (Figure 6C), indicating the ANGPTL4-regulated ARHGAP5/RhoA signaling cascade in hBMECs. Furthermore, by successfully using the RhoA specific inhibitor CCG (100 μM), we demonstrated that the ANGPTL4-induced high-expression of MYL5 could be completely blocked (Figure 6D), which further supports that ANGPTL4-induced upregulation of MYL5 depended on the ARGAP5/RhoA signaling cascade.

## 3. Discussion

Bacteria disrupting the BBB and causing meningitis rely on a multistep process that involves complex bacteria–host interactions, and different pathogenic bacteria have developed diverse strategies to assist their invasion processes [15]. As a crucial step for the development of bacterial meningitis, the complete mechanisms for the disruption of BBB induced by meningitis-causing bacteria remain to be addressed. Previously, ANGPTL4 has been shown to be involved in several biological events, such as the modulation of vascular permeability, angiogenesis, and inflammatory signaling in many other disease models [32,33]. A recent work has demonstrated a protective effect of ANGPTL4 on the BBB after ischemic stroke injury and reperfusion by thrombolysis [34]. However, this molecule has never been reported in bacterial meningitis, and the specific roles of ANGPTL4 during this pathogenic process is still unclear. In the current study, together with our previous RNA-sequencing work [30], we demonstrated in vivo and in vitro that ANGPTL4 was significantly and time-dependently upregulated in hBMECs in response to meningitic *E. coli*, suggesting that ANGPTL4 is likely involved in the process of meningitic *E. coli* interaction with the BBB. We further explored in vivo and in vitro the potential effects of ANGPTL4 on the BBB permeability and determined the influence of ANGPTL4 expression as well as its associated signaling pathway in meningitic *E. coli*-induced damage of the BBB. To our knowledge, this is the first functional demonstration and characterization of ANGPTL4 in meningitic *E. coli* interaction with the BBB, which shall further extend the current understanding of the pathogenic mechanism involved in the development of bacterial meningitis.

The alteration of intercellular tight junction is widely recognized as an important hallmark in meningitic bacterial induction of the BBB disruption, which could be mediated by a direct regulation of the tight junction proteins, as well as by an indirect inflammatory response-mediated tight junction damage [35,36,37,38,39]. However, interestingly, we demonstrated in this work that meningitic *E. coli*-caused ANGPTL4 induction of BBB disruption depended neither on direct damage of the tight junction proteins by ANGPTL4, nor the ANGPTL4-mediated proinflammatory cytokines production—by the demonstrations that all canonical tight junction proteins like ZO-1, Claudin 5, Occludin; the proinflammatory cytokines involving in BBB disruption like IFN-γ, IL-1β, TNF-α, IL-6, IL-8 [35,39]; as well as the potential endothelial activation markers like EGR-1, E-selectin, ICAM-1, and VEGFA were actually not affected in response to ANGPTL4 treatment or overexpression, which suggested that there might be a novel mechanism of ANGPTL4-mediated BBB disruption during meningitic *E. coli* infection process. Thus, here comes the question of how ANGPTL4 affects the barrier function of cells.

To further explore this, we performed the RNA-sequencing analysis in hBMECs stimulated by rANGPTL4 protein. While the sequencing identified a total of 408 DEGs in the cells upon ANGPTL4 treatment, we focused on two significantly enriched pathways involving “pathogenic *E. coli* infection” and “leukocyte transendothelial migration”. Two candidate genes, MYL5 and ARHGAP5, were finally verified to be the potential targets of ANGPTL4 treatment. 

Among these two genes, the product of MYL5 belongs to the myosin light chain (MLC) family [40], and members in this family have important regulatory roles in a wide range of cellular and physiological processes, including maintaining the normal cell cytoskeleton [41]. Previous research found that the MLC family were also the chromatin-associated nuclear proteins that participated in gene transcription [42]. Additionally, the myosin light chain kinase (MLCK)-induced phosphorylation of MLC played important roles in the cell contraction response [43]. Here in the current study, we demonstrated for the first time the significant induction of MYL5 in hBMECs in response to meningitic *E. coli* infection as well as the ANGPTL4 treatment, and the increased MYL5 led to the BBB dysfunction by affecting the cellular cytoskeleton, which was actually consistent with several previous studies showing that many members in the MLC family have been shown to be able to change the shape of endothelial cells via regulating F-actin microfilaments [41,44]. Moreover, we explored the possible pathway involving the ANGPTL4-mediated MYL5 upregulation, and, coincidentally, the second potential targets of ANGPTL4 from our RNA-sequencing results, ARHGAP5, was found to be associated. Herein, ARHGAP5 was significantly downregulated in meningitic *E. coli*-challenged hBMECs, and in ANGPTL4-treated cells as well. ARHGAP5 is a proto-oncogene that encodes Rho GTPase-activating protein 5, a main negative regulator of the Rho-dependent signaling processes which participates in multiple cellular processes such as cell adhesion, migration, invasion, and cytokinesis [31]. In contrast, the MLC proteins are reported to be the key downstream effector of RhoA signaling [41,45]. Via regulating RhoA signaling activity, the expression of ARHGAP5 in cells was shown to affect actin cytoskeleton-based stress fibers formation [31], and this actin cytoskeleton rearrangement or movement has been evidenced in our previous work to lead to alteration of cell permeability [46]. Taking all these clues together, we therefore present our hypothesis and have evidenced that meningitic *E. coli* infection of hBMECs triggered ANGPTL4-mediated ARHGAP5/RhoA signaling, which increased MYL5 expression and finally led to the dysfunction of BBB, and this ANGPTL4–ARHGAP5–RhoA–MYL5 may present another way for meningitic *E. coli* penetration of the BBB. 

**Figure 7:**
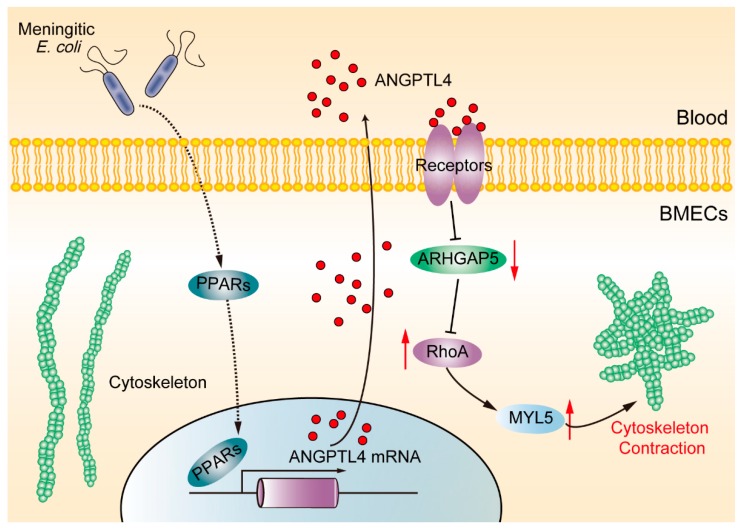
Schematic diagram of the pathways and molecules involved in ANGPTL4-mediated BBB disruption in response to meningitic *E. coli*.

In summary, we confirmed the importance of ANGPTL4 in meningitic *E. coli*-induced disruption of the BBB. We verified the PPARs pathway-mediated generation of ANGPTL4 in BMECs in response to meningitic *E. coli* challenge and demonstrated for the first time that ANGPTL4-triggered ARHGAP5/RhoA/MYL5 signaling cascade contributed to the BBB disruption by the infection (Figure 7). Characterization of these important targets involved in pathogenic bacteria–host interaction, such as ANGPTL4 herein, shall further extend and consolidate the current understanding of pathogenic mechanism during the development of bacterial meningitis and will lay important foundation for future prevention as well as therapy of the CNS disorder.

## 4. Materials and Methods 

### 4.1. Bacteria, Cells, and Culture Conditions

The pathogenic *E. coli* strain PCN033 was originally isolated from brain tissue of a diseased pig with neurological signs from Hunan province of China [47]. This isolate was subsequently evidenced to be highly pathogenic and was capable of invading hBMECs and inducing BBB disruption [16,48]. PCN033 strain was cultured on Luria–Bertani (LB) agar and/or in LB broth aerobically at 37 °C for 12 h and washed in PBS prior to the following assays unless other specified.

The hBMECs was kindly gifted by Professor Kwang Sik Kim at Johns Hopkins University School of Medicine [49] and routinely cultured in RPMI1640 medium supplemented with 10% heat-inactivated fetal bovine serum, 2-mM L-glutamine, 1-mM sodium pyruvate, vitamins, nonessential amino acids, and penicillin (100 U/ml) and streptomycin (100 μg/ml) at 37 °C under 5% CO_2_ until reaching monolayer confluence. Cells were washed with WXM medium (mixed 199 medium with F-12 medium at ratio of 1:1) and starved in serum-free medium for 16–18 h before further treatment. For bacterial challenge, the cells were infected with pathogenic *E. coli* strain PCN033 at multiplicity of infection (MOI) of 10 for 3 h.

### 4.2. Protein Isolation and Western Blotting

The hBMECs were lysed in radio immunoprecipitation assay (RIPA) buffer (Beyotime, Shanghai, China) with protease inhibitor cocktail (Beyotime) and centrifuged at 12,000 rpm for 10 min at 4 °C to remove the insoluble cell debris. The total protein concentration from cell lysates was measured with BCA protein assay kit (NCM Biotech, China) and applied to the western blot analysis with corresponding antibodies, which was performed as in a previous study [16]. The anti-ZO-1, anti-Occludin, anti-Claudin5, anti-ARHGAP5, and anti-RhoA were ordered from Abcam (Cambridge, MA, USA). The densitometry analysis was performed using ImageLab software version 5.2.1 (Bio-Rad, Hercules, CA, USA).

### 4.3. siRNA/shRNA Knocking-Down and Gene Overexpression

For siRNA knocking-down assays, siRNAs of PPARβ/δ and PPARγ were synthesized by GenePharma Biotech (Shanghai, China) and then transfected into hBMECs on 6-well plates by using an INTERFERin siRNA transfection reagent (Polyplus, Illkirch, France). After 24 h of incubation, cells were collected for the total RNA extraction as well as cDNA transcription. The efficacy of siRNA knockdown was evaluated by qPCR with primers listed in Table S2. The expression of ANGPTL4 in the PPARβ/δ and PPARγ interfered hBMECs was determined by qPCR with primers listed in Table S2. GAPDH was used as reference control for the qPCR assays, and each assay was repeated three times independently.

For shRNA assays, the shRNA of MYL5 (synthesized by GenePharma Biotech, Shanghai, China) was transfected into hBMECs on 6-well plates by using Invitrogen Lipofectamine^TM^ 3000 Transfection Regent (Thermo Scientific, Waltham, MA, USA). After 24 h of incubation, cells were cultured and passaged in a medium supplemented with G418 (Thermo Scientific) for approximately 21 days to screen and yield the stable shMYL5 hBMECs.

For ANGPTL4 overexpression, the overexpression plasmid pcDNA-ANGPTL4 was constructed by PCR-cloning the ANGPTL4 encoding gene, with Forward primer 5′-GGGGTACCATGAGCGGTGCTCCGAC-3′ (KpnI) and Reverse primer 5′-CCGCTCGAGCTAGGAGGCTGCCTCTGCT-3′ (XhoI), into the pcDNA3.1(+) vector. The cloned plasmid was then transfected into hBMECs using the jetPRIME^®^ DNA transfection reagent (Polyplus, France) following the instructions. After 24 h of incubation, cells were collected for total RNA extraction and transcription, and qPCR was used to determine the expression level of the ANGPTL4 encoding gene. The vehicle plasmid pcDNA3.1(+) was synchronously transfected as the control.

### 4.4. Cell Infection and Treatments

The confluent hBMECs monolayer in 6-cm dishes (Corning, NY, USA) were infected by PCN033 at MOI of 10 for 0 h, 1 h, 2 h, and 3 h. In some assays, cells were pretreated with the PPARβ/δ inhibitor GSK3787 (Selleck Chemicals, Houston, TX, USA), PPARγ inhibitor T0070907 (Selleck Chemicals), or RhoA inhibitor CCG1423 (Selleck Chemicals) for 3 hours prior to the infection, or incubated with 100 ng human rANGPTL4 protein (R&D Systems, Minneapolis, MN, USA) for 24 h. Cells were then collected for total RNA extraction and cDNA transcription. The expression of ANGPTL4, PPARβ/δ, PPARγ, EGR-1, E-selectin, ICAM-1, VEGFA, MYL5, ARHGAP5, and RhoA were determined by qPCR with primers listed in Table S2. GAPDH was used as the reference control. Each qPCR assay was performed in triplicates. Total protein expression of ZO-1, Claudin5, Occludin, ARHGAP5, and RhoA was determined by Western Blotting. GAPDH or β-actin was detected as the loading control.

### 4.5. MTT Assay

The putative cytotoxicity of rANGPTL4 on hBMECs was determined by using the MTT Cell Proliferation and Cytotoxicity Assay Kit (Sigma-Aldrich, MO, USA) following the manufacturers’ instructions. Briefly, 2 × 10^4^ cells of hBMECs were seeded in quintuplicates in 96-well plates and cultured overnight. The human rANGPTL4 protein was added into the wells at the final concentrations from 1 ng/ml to 100 ng/ml in the medium. A total of 0.5% MTT solution was subsequently added into each of the wells and the cells were continuously incubated at 37 °C under 5% CO_2_ for another 4 h. Methanol solvent at a final concentration of 0.5% was used as control. The supernatant was removed and Dimethyl sulfoxide (DMSO) was then added to resolve the pellet. The plate was finally subjected to test OD560 values in a Bio-Rad reader (Bio-Rad, CA, USA).

### 4.6. Electric Cell-Substrate Impedance Sensing (ECIS)

The putative effect of rANGPTL4 on the barrier function of hBMECs was determined by the ECIS technology. Briefly, approximately 7 × 10^4^ cells of hBMECs were seeded on collagen-coated, gold-plated electrodes in 96-well chamber slides (96W1E+), linked to ECIS Zθ equipment (Applied BioPhysics, NY, USA), and continuously cultured until confluence was reached. The TEER was monitored to reflect the formation of the barrier, as described previously [50]. After stable maximal TEER was reached, rANGPTL4 protein (final concentrations at 1 ng/ml, 10 ng/ml, 50 ng/ml, and 100 ng/ml) was added into the cells, and the TEER changes were automatically recorded by the ECIS system. Likewise, this ECIS system was also applied to test the putative effect of MYL5 shRNA knocking-down on the barrier function of hBMECs. 

### 4.7. Flow Cytometry

The putative effect of rANGPTL4 on the apoptosis of hBMECs was determined by flow cytometry with a commercial Annexin V-FITC Apoptosis Detection Kit (Beyotime, China) following the manufacturers’ instructions. Briefly, confluent hBMECs in 6-well plates were treated with rANGPTL4 (100 ng/ml) or PBS, and then were digested with trypsin (without EDTA), washed with PBS, and centrifuged at 180× g for 5 min. After suspension with the binding buffer in the kit, cells were transferred into tubes and treated with AnnexinV-PI provided in the kit, and finally detected with BD FACSVerse™ flow cytometer (BD Biosciences, NJ, USA).

### 4.8. Electrochemiluminescence (ECL) Assay

To determine the potential effect of ANGPTL4 on the generation of cytokines, the hBMECs monolayer confluence were incubated with 100 ng/ml human rANGPTL4 protein for 24 h. As the control, cells were synchronously treated with PBS. After the treatment, the culture supernatant were collected and subjected to the quantitation of multiple cytokines, including IFN-γ, IL-1β, TNF-α, IL-6, and IL-8, by using the ECL assay according to the manufacturer’s instructions (Meso Scale Discovery, Rockville, MD, USA). Each treatment contained three replicates for the ECL assays.

### 4.9. Immunofluorescence

The immunofluorescence was performed to observe the F-actin alteration by staining with Actin-Traker Green (Beyotime). Briefly, the monolayer cells were fixed in formalin solution for 10 min and washed with PBS containing 0.1% TritonX-100 for three times. Cells were then stained with Actin-Traker Green at 1:100 dilution in PBS containing 5% BSA and 0.1% TritonX-100 for 1 h. After three times wash in PBS containing 0.1% TritonX-100, the cells were stained with DAPI (Beyotime, China) and were finally observed on a Zeiss MIC-SYSTEM (Carl Zeiss, Germany).

### 4.10. RNA-Sequencing and Analyses

The hBMECs monolayer confluence were incubated with 100 ng/ml human rANGPTL4 protein for 24 h. For the control group, cells were treated with PBS. Total RNAs from the treated cells (three biological replicates per condition) were extracted by using TRIzol reagent (Invitrogen, Carlsbad, CA, USA) and evaluated by the NanoPhotometer^®^ spectrophotometer from IMPLEN (LA, CA, USA), and then sent to Personalbio company (Shanghai, China) for RNA purity and integrity analyses as well as sequencing. Briefly, a total amount of 3 μg RNA per sample was used as input material for the RNA sample preparations. Sequencing libraries were generated using NEBNext^®^ Ultra™ RNA Library Prep Kit for Illumina^®^ (New England Biolabs, Beverly, MA, USA) following manufacturers’ recommendations and index codes were added to attribute sequences to each sample. The library preparations were sequenced on an Illumina Hiseq 2000 platform and 100-bp paired-end reads were generated. Raw data (raw reads) of fastq format were firstly processed through in-house perl scripts. In this step, clean data (clean reads) were obtained by removing reads containing adapter, reads containing ploy-N, and low-quality reads from raw data. At the same time, Q20, Q30, and GC content of the clean data were calculated. All the downstream analyses were based on the clean data with high quality. Differentially expressed genes (DEGs) were identified using the DESeq R package (1.10.1). The resulting *p*-values were adjusted using the Benjamini and Hochberg’s approach for controlling the false discovery rate. Genes with an adjusted *p*-value < 0.05 found by DESeq were assigned as differentially expressed. Gene Ontology (GO) enrichment analysis of DEGs was implemented by the GOseq R package, in which gene length bias was corrected. GO terms with corrected *p*-value less than 0.05 were considered significantly enriched by differential expressed genes. KOBAS software was used to test the statistical enrichment of DESeq in KEGG pathways.

### 4.11. Animal Tests

The current study was carried out in accordance with the guidelines established by the China Regulations for the Administration of Affairs Concerning Experimental Animals (1988) and Regulations for the Administration of Affairs Concerning Experimental Animals in Hubei Province (2005) (Animal Welfare Assurance No.190918). All procedures and handling techniques were approved by the Committee for Protection, Supervision, and Control of Experiments on Animals guidelines at Huazhong Agriculture University (Permit No. SYXK 2018-0070). All efforts were made to treat the experimental animals in this study ethically and to minimize suffering. A total of 50 mice were randomly divided into ten groups (group I to X) and each group contained 5 mice. Mice in groups I to V were treated with PBS, 10 ng, 20 ng, 50 ng, and 100 ng of the rANGPTL4 protein through the tail vein, respectively. After 24 hours of injection, each mouse was given an injection of 500 μl Evans blue dye (Sigma-Aldrich, CA, USA) through tail vein for another 20 min. Mice in groups VI to X were pretreated with PBS (200 μl), PBS (200 μl), GSK3787 (10mg/kg), T0070907 (1.5 mg/kg), and GSK3787 (10 mg/kg) plus T0070907 (1.5 mg/kg), respectively. After 4 hours of pretreatment, each mouse in group VII to X was challenged with meningitic *E. coli* PCN033 in PBS at 1.0 × 10^8^ CFU/mouse through the tail vein. After 8 hours of infection, each mouse in all groups received the injection of 500 μl Evans blue through tail vein for 20 min. Mice were subsequently anesthetized and then subjected to cardiac perfusion. Mouse brains were finally collected to observe the alteration of BBB permeability.

### 4.12. Statistical Analysis

Significance of the differences between each group was analyzed by two-way analysis of variance (ANOVA) embedded in GraphPad Prism version 6.0 and Student’s t-test. Data were presented as mean + standard deviation (mean + SD), and the statistical significance levels were set as *p*-value < 0.05 (*, significant), *p*-value < 0.01 (**, extremely significant), and *p*-value < 0.001 (***, extremely significant).

## Supplementary Materials

The following are available online at https://www.mdpi.com/2076-0817/8/4/254/s1, Figure S1: The expression of several host molecules probably involved in BBB disruption in response to rANGPTL4. Figure S2: The expression of several host molecules probably involved in BBB disruption in ANGPTL4-overexpressed hBMECs. Table S1: Enriched KEGG Pathway (*p* < 0.05). Table S2: The list of primers for qPCR.
